# Brain wave classification using long short-term memory network based OPTICAL predictor

**DOI:** 10.1038/s41598-019-45605-1

**Published:** 2019-06-24

**Authors:** Shiu Kumar, Alok Sharma, Tatsuhiko Tsunoda

**Affiliations:** 1 Institute for Integrated and Intelligent Systems, Griffith University, Brisbane, QLD-4111 Australia; 2 Department of Medical Science Mathematics, Medical Research Institute, Tokyo Medical and Dental University, Tokyo, Japan; 3 Laboratory for Medical Science Mathematics, RIKEN Center for Integrative Medical Sciences, Yokohama, Kanagawa Japan; 4 The University of the South Pacific, Suva, Fiji; 5 Fiji National University, Suva, Fiji; 6 CREST, JST, Tokyo, 102-8666 Japan

**Keywords:** Electrical and electronic engineering, Neural decoding, Biomedical engineering

## Abstract

Brain-computer interface (BCI) systems having the ability to classify brain waves with greater accuracy are highly desirable. To this end, a number of techniques have been proposed aiming to be able to classify brain waves with high accuracy. However, the ability to classify brain waves and its implementation in real-time is still limited. In this study, we introduce a novel scheme for classifying motor imagery (MI) tasks using electroencephalography (EEG) signal that can be implemented in real-time having high classification accuracy between different MI tasks. We propose a new predictor, OPTICAL, that uses a combination of common spatial pattern (CSP) and long short-term memory (LSTM) network for obtaining improved MI EEG signal classification. A sliding window approach is proposed to obtain the time-series input from the spatially filtered data, which becomes input to the LSTM network. Moreover, instead of using LSTM directly for classification, we use regression based output of the LSTM network as one of the features for classification. On the other hand, linear discriminant analysis (LDA) is used to reduce the dimensionality of the CSP variance based features. The features in the reduced dimensional plane after performing LDA are used as input to the support vector machine (SVM) classifier together with the regression based feature obtained from the LSTM network. The regression based feature further boosts the performance of the proposed OPTICAL predictor. OPTICAL showed significant improvement in the ability to accurately classify left and right-hand MI tasks on two publically available datasets. The improvements in the average misclassification rates are 3.09% and 2.07% for BCI Competition IV Dataset I and GigaDB dataset, respectively. The Matlab code is available at https://github.com/ShiuKumar/OPTICAL.

## Introduction

Brain-computer interface (BCI) has become a hot topic of research as it is increasingly being used in gaming applications^1^ and in stroke rehabilitation^2–7^ for translating the brain signals of the imagined task into intended movement of the limb that has been paralyzed. For example, BCI controlled wheel-chairs^2,8,9^ are being developed to enable people with disabilities to maneuver around the house and perform basic tasks. Moreover, BCI research is also being carried out to detect in advance that a person is going to suffer from a seizure attack so that they can be informed in order to prevent accident or serious injuries^10–12^. Electroencephalography (EEG) signal obtained using non-invasive sensors have been widely used^12,13^ for these purposes due to its low cost, easy to use and that it does not require any surgery as required by invasive sensors. BCI using non-invasive sensors are approaching their required technological advancements and translate neural activities into meaningful information that can be used to drive activity-dependent neuroplasticity or rehabilitation robots. Although some promising results have been achieved, BCI for rehabilitation is still a new and emerging field. Therefore, being able to classify the different tasks with greater accuracy using the EEG signal will not only be beneficial for gaming and rehabilitation but also help in better detection of diseases or abnormal behaviors such as seizure^12,14^, sleep apnea^15^, sleep stages^16,17^, and drowsiness^18^ detection. Thus, developing a BCI system that can classify different types of EEG signals with high accuracy is highly desirable.

Common spatial pattern has been widely used for extracting the features from EEG signals for classification. However, the responsive frequency range varies from subject to subject and for this reason subject dependent BCI’s^19–25^ are being mostly proposed. A poorly selected frequency band may contain unwanted or redundant information and will degrade the performance of the overall system. The selection of the frequency bands plays a key role in extracting significant features and manually tuning the filters will be a difficult task. To tackle this problem, many subject-dependent approaches utilizing multiple frequency bands have been proposed^21,26–36^. These methods use multiple filter bands to filter the signal into different sub-bands and then utilize CSP for extracting the features. Some approaches proposed different methods of selecting the best sub-bands^21,24,32,34^ while other approaches considered various feature selection techniques^20,28,31,35–37^ using all sub-bands to achieve promising results. While appropriately using multiple sub-bands helped achieve improved performance, it also increased the computation complexity of the system^38^. Few researchers have also considered directly improving the CSP algorithm^39–43^ for better performance. Other methods that have been proposed use wavelet packet decomposition^23^, empirical mode decomposition^19,29^, Riemannian tangent space mapping^22,44^, artificial neural networks^40,45,46^ and deep learning^47,48^.

Deep learning has recently gained widespread attention in the field of signal processing. However, it has not been fully explored for EEG signal classification. In this study, we focus on subject-dependent approach and propose an Optimized CSP and LSTM based predictor named OPTICAL. An LSTM network is a recurrent neural network consisting of LSTM layers having the ability to selectively remember important information for a longer period and is mostly used for sequence prediction. As reported in our previous works^30^, to keep the computational complexity of the system low, OPTICAL uses a single Butterworth band-pass filter with cutoff frequencies of 7–30 Hz. Promising 10 × 10-fold cross-validation results have been obtained using OPTICAL, which has been evaluated using the BCI Competition IV dataset 1^49^ and GigaDB dataset^50^. OPTICAL showed improvement in the classification performance (achieving average misclassification rate of 17.48% and 31.81% for BCI Competition IV dataset 1 and GigaDB dataset, respectively) and can be beneficial in developing improved BCI systems for rehabilitation. Apart from this, if applied appropriately, it might also help detect seizure, sleep apnea and sleep stages with greater accuracy. The results obtained are superior compared to other competing methods. Thus, we have shown that appropriately using LSTM network can help develop improved BCI systems. Almost all the related works^19,20,23,26,31,34,35,51,52^ considered classification of MI tasks, which were limited to binary class MI EEG signal classification problem. However, it should be noted that real-time EEG signal contains noise and other activities (such as eye blinking, eyeball movement up/down, eyeball movement left/right, jaw clenching and head movement left/right), referred to as non-task related EEG signals. Thus, it is important to show that the proposed approach will be able to perform well if the implementation takes place in real-time. Therefore, we utilize the rest-state and non-task related EEG signals to show that the proposed method will perform well for real-time classification. For this purpose, we have utilized the one-versus-rest approach (as using the one-versus-rest approach yields substantially better results than using the multi-class classification) for classification of the multi-class MI tasks using the conventional CSP algorithm. The GigaDB dataset, which also provides the recordings for the rest-state and other non-tasks related signals, has been used to show the effectiveness of OPTICAL for real-time implementation. For real-time implementation, we achieved an average misclassification rate of 17.78% over 52 subjects using GigaDB dataset.

## Materials and Methods

In this study, we propose a machine learning-based optimized predictor that combines the LSTM network with CSP for the classification of EEG signals named OPTICAL. The following sections describe the publically available benchmark datasets that are used to evaluate the performance of OPTICAL. A detailed overview of the OPTICAL predictor is also presented.

### Benchmark dataset 1 – BCI competition IV dataset 1

The BCI competition IV dataset 1^53^ is a publically available dataset provided by the Berlin BCI group. This dataset contains EEG recordings of seven healthy subjects performing two MI tasks without any feedback. Out of the seven subjects, the data for subjects *c*, *d* and *e* were artificially generated. The two MI tasks performed by each subject were selected from the left hand, right-hand and foot MI tasks. BrainAmp MR plus amplifiers and an Ag/AgCl electrode caps were used to acquire the EEG recordings. 59 channels were used to record the data at a sampling frequency of 1000 Hz. A down-sampled version at 100 Hz, which is also made available, has been used in this study consisting of 200 trials for each subject having an equal number of each trial. A detailed description of the dataset can be found at the given reference.

### Benchmark dataset 2 – GigaDB dataset

The GigaDB dataset^50^ is a publically available dataset that has been published recently. It consists of EEG recordings of the left hand and right-hand MI tasks of 52 healthy subjects out of which 19 were female subjects. All subjects were right-handed except for subject’s s20 and s33 that were both handed. The EEG data were acquired using 64 Ag/AgCl active electrodes at a sampling rate of 512 Hz. The electrodes were placed based on the international 10–10 system. Apart from the left and right-hand MI tasks, non-task related EEG data such as eye blinking, eyeball movement up/down, eyeball movement left/right, head movement, jaw clenching, and resting state were also recorded. The results of a psychological and physiological questionnaire and EMG data are also made available. However, these have not been used in this study. 100 or 120 trials of each task-related (left and right-hand) MI EEG signal for each subject were recorded. The dataset contains a combination of well-discriminated data (38 subjects) and less-discriminative data. For a detailed description of the dataset, refer to the published dataset description^50^.

### Preprocessing

In this study, we have taken a two seconds window 0.50 seconds after the visual cue was presented to perform the MI tasks. This is the same as done in other related works^21,22,26,38,50^. Common average referencing has been applied to all the trials. Each trial is then filtered using a Butterworth bandpass filter having passband cutoff frequencies of 7 and 30 Hz. This preprocessed data is utilized for further processing.

### The proposed predictor (OPTICAL)

**Figure 1:**
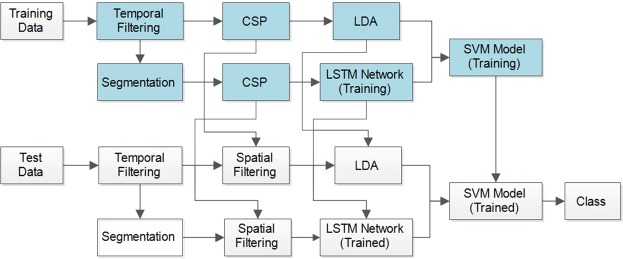
The framework of the proposed predictor, OPTICAL.

**Figure 2:**
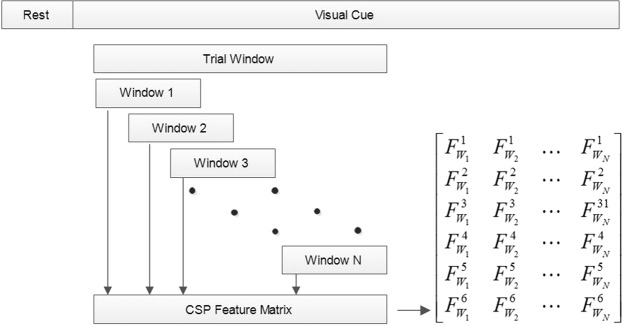
Performing segmentation and obtaining the feature matrix.

The framework of the proposed subject-dependent predictor, OPTICAL, is shown in Fig. 1. The predictor is named OPTICAL as it combines CSP and LSTM, and the LSTM network is optimized using Bayesian optimization. As can be seen from Fig. 1, two sets of CSP spatial filters are learned by the predictor. One set of spatial filters are directly learned from the trials of the training data after temporal filtering. The variance based CSP features are extracted from these spatially filtered data and linear discriminant analysis (LDA) is then applied to these features to obtain a one-dimensional feature. The second set of CSP spatial filters is learned from the combined data that is obtained after the segmentation of each of the trials from the training data as shown in Fig. 2. Each trial data is broken down into smaller parts by taking a smaller window of length *l* sample points with an overlap of *t* sample points, resulting in *N* segments being obtained from each trial. These *N* segments obtained from each of the training trials are used to learn the second set of CSP spatial filters. All the segments are then spatially filtered using this set of spatial filters. The variance based features of each segment from a single trial are computed and a feature matrix as shown in Fig. 2 is formed. In the feature matrix, ${F}_{{W}_{j}}^{i}$ represents the *i*-th feature obtained from the *j*-th windowed segment of the respective trial. This is repeated for all the trials to obtain the feature matrix of all the trials, which becomes the input to the LSTM network.

The inputs to the LSTM network are the feature matrices of all the training trials that are used to train the network. Each of these feature matrices represents the sequential input of each trial. The LSTM network thus uses these sequential inputs to train the network. The LSTM network used in this study consists of the sequence input layer, two consecutive LSTM layers having 100 and 20 hidden units in first and second LSTM layers respectively, a fully connected layer and a regression output layer. Since the output layer is a regression layer, the output is a one-dimensional vector. Thus, the one-dimensional feature vector obtained after performing LDA is concatenated with the output feature vector of the LSTM network and fed to a support vector machine (SVM) classifier. The SVM model is trained using these features of the training data. In the training phase, the LSTM network hyper-parameters are optimized using Bayesian optimization and the optimized hyper-parameters are then used in the test phase. A test trial is first filtered using the band-pass filter. The filtered test trial then undergoes 2 different processes as in training phase. In the first process the filtered test data is filtered using the corresponding spatial filter learned during the training phase to obtain the CSP variance based features and the dimensionality of the features reduced using the corresponding LDA vector learned during training phase. The resulting output becomes one of the features for the trained SVM model. In the second process, the filtered test trial is segmented and the segmented data is spatially filtered using the corresponding spatial filter learned during the training phase. CSP variance based features are extracted and the feature matrix of the test trial is formed, which is fed to the trained LSTM network. The regression output of the LSTM network becomes the second feature for the trained SVM model. Finally, using these two features the SVM model predicts the class/task the test trial belongs to. This procedure is repeated for each of the test trials. It should be noted that the proposed approach is subject-dependent. We take a subject at a time and then we split this subjects data into training and test set using 10-fold cross-validation scheme. Then we use training set to train the model while test set is used to evaluate the model. This procedure is repeated 10 times resulting in 10 × 10-fold cross-validation scheme. The misclassification rates obtained from 10 × 10-fold cross-validation scheme are averaged and this result is reported as the misclassification rate for the subject. The above process is repeated for each of the subjects in order to obtain their respective misclassification rate. The average misclassification rate for a dataset is calculated by averaging the misclassification rates over all the subjects in the dataset.

#### Common spatial pattern

The common spatial pattern has been widely used for EEG signal processing. It projects the data into a new time-series where the variance of one class is minimized while that of the other class is maximized. Thus the variance based CSP features are utilized for classification. A detail description of the CSP algorithm can be found in our previous work^30^. Once the spatial filter *W* is determined using the training data, an EEG trial *E* can be filtered using equation (1) to obtain the spatially filtered signal, *Z*. *T* in equation (1) represents the transpose operator. The features of a single trial can then be obtained using equation (2). The CSP variance based features of all data can be obtained following these procedures.

1 $$Z={W}^{T}E$$

2 $$y=\,\mathrm{log}(\mathrm{var}(Z))$$

#### Long short-term memory (LSTM) network

Deep learning has been gaining widespread attention and performing well compared to other conventional methods in many applications. One of the deep learning networks is the recurrent neural network and a recurrent neural network having LSTM layers is usually referred to as an LSTM network. The LSTM network has been seen to be more effective than the feed-forward neural networks and recurrent neural networks (not containing any LSTM layer) in terms of sequence prediction due to their ability to selectively remember important information or values for a longer period of time. An LSTM network is usually used for processing and classifying or predicting time-series or sequence data. In this study, we propose a novel idea to apply LSTM for EEG signal processing. A sliding overlapping window is applied to each trial to obtain a feature matrix in the form of sequence data, which is used as the sequence input to the LSTM network. In general, the LSTM architecture comprises of a memory cell, an input gate, a forget gate and an output gate. The memory cell of the LSTM layer stores or remembers values (states) for either long or short times. On the other hand, the degree to which a new information or value flows into the cell of an LSTM layer is controlled by the input gate, the degree to which an information or value remains in the cell of the LSTM layer is controlled by the forget gate while the degree to which information or value stored in the cell of the LSTM layer is utilized for computing the output activation is controlled by the output gate. OPTICAL utilizes a recurrent neural network comprising of two LSTM layers. A detailed explanation of the LSTM network can be found in Supplement 1 and other related works^54,55^.

#### Selection of the hyper-parameters for the LSTM network

**Figure 3:**
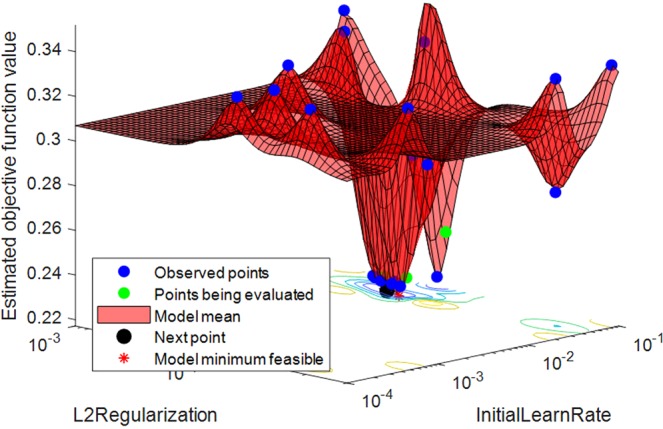
Determining the best feasible hyper-parameters of LSTM network using Bayesian optimization.

The performance of the LSTM network depends on a number of hyper-parameters such as the network size, initial learn rate, learn rate schedule (which has hyper-parameters such as learn rate drop factor and learn rate drop period), momentum and L2 regularization. The network size selected in this work is explained in the discussion section. The parameter selection of other hyper-parameters is carried out using Bayesian optimization technique. It was noted that using piecewise schedule in the optimization process as the learn rate schedule did not perform well and thus we have used the default settings. This could be due to the number of training samples not being large enough, which is mostly the case for BCI applications. We have used the Stochastic gradient descent momentum, which utilizes a contribution proportional to the previous iterations update for the current update. The initial learn rate and L2 regularization depends on the data and the network used or selected. Therefore, to select the best initial learn rate and L2 regularization parameters for achieving optimal results we have employed Bayesian optimization technique. The range for the initial learn rate and L2 regularization were set to [1E-4, 1E-1] and [1E-5, 1E-3], respectively. These hyper-parameters were set around the default hyper-parameter values. 10-fold cross-validation has been used on the training data during the Bayesian optimization for selecting the best parameters. Figure 3 shows the effect of selecting different values for the initial learn rate and L2 regularization parameters for one of the trials runs of subject *a* of BCI competition IV dataset 1. It shows how the Bayesian optimization technique can determine the best feasible values for these two hyper-parameters and justifies the need for optimizing the network parameters.

### Support Vector Machine (SVM)

SVM is a supervised learning technique that can be utilized for both classification and regression. The SVM algorithm finds a hyperplane that maximizes the separation of the support vectors. In this study, we have used an SVM classifier with radial basis kernel function. Use of kernel function allows mapping of non-linear data to a higher dimension where the data are linearly separable.

### Performance measures

It is very important to evaluate the performance of the predictor that is designed or proposed. The performance measures used to evaluate the performance of OPTICAL are the misclassification rate, sensitivity, specificity, and Cohen’s kappa index (κ). The misclassification rate shows the percentage of trials in the test data that have been incorrectly classified. The sensitivity shows the ability of the classifier or predictor to correctly classify the positive trials while specificity shows the ability of the classifier or predictor to classify the negative trials correctly. Cohen’s kappa index is a statistical measure that is used to assess the reliability of the classifier or predictor. These performance measures are calculated using equations (3–6), where TP is the true positives, TN is the true negatives, FP is the false positives, FN is the false negatives, *p*_*e*_ is the chance of agreement that is expected and *p*_*a*_ is actual percentage of agreement. Lower values of misclassification rate and higher values of sensitivity, specificity and Cohen’s kappa index are preferred.

3 $${\rm{misclassification}}\,\,{\rm{rate}}=\frac{FP+FN}{TP+TN+FP+FN}$$

4 $${\rm{sensitivity}}=\frac{TP}{TP+FN}$$

5 $${\rm{specificity}}=\frac{TN}{TN+FP}$$

6 $$\mathrm{Cohen}\text{'}s\,{\rm{kappa}}\,{\rm{index}}=\frac{{p}_{a}-{p}_{e}}{1-{p}_{e}}$$

## Results

To make a fair comparison between OPTICAL and other competing methods, we have evaluated all the methods using 10 × 10-fold cross-validation scheme. In this scheme, the data is divided into 10 segments where 9 segments are used as training set while the remaining one segment is used as test set. This procedure is repeated by taking a new segment each time for testing until all subsets are used exactly once. The whole procedure is then repeated 10 times and the results are averaged. All results reported in this study are obtained using this procedure. We have compared OPTICAL with other competing methods such as the conventional CSP approach, the discriminative filter bank CSP (DFBCSP)^34^ approach, the spatial-frequency-temporal optimized feature sparse representation-based classification (SFTOFSRC) approach^20^, and the sparse Bayesian learning of filter banks (SBLFB)^26^ approach. For the conventional CSP approach, a wide band-pass filter having cutoff frequencies of 4–40 Hz has been used. Six spatial filters have been used in all the methods to make a fair comparison between all the methods. Matlab running on a personal computer at 3.3 GHz (Intel(R) Core(TM) i7) has been used for all processing in this study.

### Comparison of results with competing methods

A comparison of the misclassification rate together with the standard deviation of OPTICAL and other competing methods for BCI competition IV dataset 1 and the GigaDB dataset are given in Tables 1 and 2, respectively. The last column in Table 2 shows the misclassification rate of the proposed OPTICAL predictor for real-time implementation and is discussed later. The lowest value of the misclassification rate for each subject is highlighted in bold. OPTICAL* is the proposed OPTICAL predictor without parameter optimization of the LSTM network using Bayesian Optimization. As can be seen from Tables 1 and 2, our proposed OPTICAL predictor outperforms all other competing methods. It achieved an improvement of 3.09% and 2.07% compared to the second best performing method (SBLFB) on BCI competition IV dataset 1 and GigaDB dataset, respectively. 3 out of 7 subjects of BCI competition IV dataset 1 and 17 out of 52 subjects of GigaDB dataset achieved the lowest misclassification rate using OPTICAL. This is the highest number achieved compared to other methods that had second highest number of subjects with lowest misclassification rates (OPTICAL*: 3 out of 7 subjects for BCI competition IV dataset 1 (our proposed predictor without optimizing LSTM network hyper-parameters) and SBLFB: 11 out of 52 subjects for GigaDB dataset). All methods were able to correctly classify all the trials of subject 50 of GigaDB dataset, which may be probably due to subject 50 having a very good quality of the trials. It is also worth mentioning that OPTICAL*, our proposed predictor without parameter optimization of the LSTM network was able to achieve the lowest average misclassification rate compared to other competing methods. However, optimizing the hyper-parameters of the LSTM network for each subject resulted in further reduction of the average misclassification rate and thus has been utilized in this work.

**Table 1:** Misclassification rate (%) of different methods evaluated using BCI competition IV dataset 1.

Subject	CSP	DFBCSP	SBLFB	SFTOFSRC	OPTICAL*	OPTICAL
a	18.00 ± 9.53	16.80 ± 7.81	19.10 ± 9.73	30.67 ± 11.50	14.30 ± 7.49	**12.68** ± **9.71**
b	50.80 ± 9.86	42.90 ± 9.75	41.50 ± 11.12	45.83 ± 8.91	41.70 ± 11.85	**38.33** ± **9.94**
c	48.90 ± 9.70	35.20 ± 8.51	33.20 ± 12.52	45.00 ± 10.42	34.10 ± 10.18	**28.17** ± **11.02**
d	35.30 ± 10.27	23.50 ± 8.41	**11.50** ± **7.91**	32.93 ± 10.96	14.70 ± 9.66	11.83 ± 7.60
e	30.70 ± 11.29	18.30 ± 8.84	11.60 ± 6.88	40.33 ± 13.13	**10.90** ± **6.28**	11.00 ± 6.22
f	31.30 ± 11.10	14.30 ± 8.57	21.20 ± 11.97	31.83 ± 11.33	**13.50** ± **6.41**	14.17 ± 7.66
g	7.60 ± 6.57	9.00 ± 5.05	5.90 ± 5.41	20.00 ± 10.59	**5.60** ± **4.59**	6.17 ± 5.03
Average	31.80 ± 9.76	22.86 ± 8.13	20.57 ± 9.36	35.21 ± 10.98	19.26 ± 8.07	**17.48** ± **8.17**

**Table 2:** Misclassification rate (%) of different methods evaluated using GigaDB dataset.

Subject	CSP	DFBCSP	SBLFB	SFTOFSRC	OPTICAL*	OPTICAL	Real-time
1	27.30 ± 12.91	37.20 ± 9.54	37.20 ± 9.85	40.17 ± 10.63	20.70 ± 8.33	**20.00** ± **9.37**	15.00 ± 9.05
2	49.00 ± 9.31	50.20 ± 11.56	**44.10** ± **12.65**	58.33 ± 8.64	45.70 ± 11.47	47.67 ± 12.37	23.25 ± 4.26
3	11.40 ± 6.70	33.00 ± 10.15	8.40 ± 7.25	11.50 ± 6.97	8.60 ± 6.15	**6.00** ± **5.78**	2.75 ± 2.19
4	39.10 ± 9.41	49.40 ± 13.46	**19.40** ± **8.84**	20.83 ± 10.99	24.10 ± 8.67	22.00 ± 10.05	14.50 ± 5.50
5	1.00 ± 2.02	1.30 ± 2.22	1.00 ± 2.02	**0.50** ± **1.53**	0.90 ± 1.94	1.00 ± 2.03	0.75 ± 1.21
6	20.20 ± 9.31	20.20 ± 8.02	17.50 ± 8.16	20.67 ± 6.40	**16.60** ± **7.66**	17.67 ± 7.74	8.00 ± 6.32
7	50.42 ± 7.68	54.67 ± 10.02	**45.17** ± **8.96**	48.33 ± 10.92	47.75 ± 11.33	47.22 ± 7.92	31.67 ± 7.20
8	49.60 ± 11.20	55.00 ± 11.78	53.90 ± 11.97	58.00 ± 9.43	**43.80** ± **11.45**	44.33 ± 11.65	24.00 ± 4.28
9	49.50 ± 9.77	49.42 ± 11.67	44.33 ± 9.92	44.44 ± 9.75	43.75 ± 10.95	**43.60** ± **10.07**	27.50 ± 4.89
10	42.70 ± 11.62	58.30 ± 8.84	36.40 ± 10.05	48.00 ± 9.06	30.90 ± 11.77	**27.00** ± **10.31**	14.25 ± 6.02
11	45.80 ± 9.55	**41.70** ± **9.98**	49.80 ± 10.15	45.33 ± 7.98	49.30 ± 11.20	42.67 ± 9.89	26.00 ± 6.69
12	**31.00** ± **9.85**	42.30 ± 8.82	37.40 ± 10.26	45.00 ± 10.51	34.50 ± 10.61	32.83 ± 8.97	15.75 ± 7.55
13	**11.10** ± **5.92**	46.30 ± 9.57	11.50 ± 8.03	18.33 ± 8.64	14.60 ± 7.75	15.50 ± 8.24	9.00 ± 2.93
14	4.80 ± 4.28	35.60 ± 10.63	5.20 ± 5.34	6.17 ± 5.20	4.70 ± 4.56	**3.50** ± **3.51**	1.50 ± 2.42
15	49.90 ± 10.47	51.80 ± 11.94	**33.20** ± **11.77**	48.83 ± 13.75	43.00 ± 14.29	35.33 ± 11.29	18.25 ± 7.91
16	51.50 ± 11.92	**48.30** ± **11.00**	51.60 ± 10.02	51.33 ± 10.25	52.10 ± 10.26	52.50 ± 11.89	26.75 ± 6.67
17	51.80 ± 8.68	48.80 ± 9.88	**47.20** ± **10.79**	47.50 ± 10.06	50.20 ± 12.78	50.33 ± 9.46	24.00 ± 4.12
18	49.00 ± 11.61	51.90 ± 12.81	**41.10** ± **11.31**	50.83 ± 9.83	49.30 ± 8.75	46.67 ± 8.34	24.00 ± 5.55
19	45.50 ± 11.35	40.20 ± 9.20	**35.40** ± **10.64**	47.00 ± 12.36	42.90 ± 10.79	42.83 ± 9.16	22.25 ± 5.95
20	36.20 ± 9.61	42.70 ± 11.83	48.70 ± 10.73	47.17 ± 11.12	28.30 ± 10.67	**27.17** ± **10.14**	12.00 ± 4.97
21	45.70 ± 11.25	38.30 ± 10.53	35.20 ± 10.83	40.33 ± 9.73	40.20 ± 10.15	**34.00** ± **11.33**	16.75 ± 6.46
22	45.70 ± 10.93	45.70 ± 9.69	43.40 ± 11.71	44.00 ± 11.33	46.60 ± 9.71	41.33 ± 10.08	20.75 ± 3.55
23	32.60 ± 9.75	32.50 ± 10.66	25.40 ± 7.06	24.17 ± 9.11	19.80 ± 9.15	15.83 ± 8.31	9.75 ± 3.22
24	53.30 ± 11.41	54.50 ± 12.71	49.40 ± 9.18	50.50 ± 9.94	46.90 ± 10.97	39.33 ± 10.23	23.25 ± 5.14
25	56.70 ± 10.72	53.00 ± 12.78	**46.90** ± **11.47**	47.67 ± 10.73	49.00 ± 9.74	47.00 ± 14.18	23.00 ± 4.97
26	4.20 ± 4.21	4.30 ± 3.91	3.30 ± 3.73	5.00 ± 3.94	3.70 ± 4.02	**3.17** ± **3.34**	1.50 ± 1.75
27	52.50 ± 11.21	**44.90** ± **11.72**	51.00 ± 10.50	46.50 ± 11.00	57.00 ± 10.40	55.33 ± 9.99	28.50 ± 5.30
28	20.30 ± 8.60	24.80 ± 7.49	21.00 ± 8.08	24.17 ± 10.35	19.50 ± 8.28	**19.17** ± **4.93**	12.00 ± 2.58
29	54.80 ± 9.95	53.00 ± 11.87	57.70 ± 10.46	**52.00** ± **10.55**	56.30 ± 11.15	57.00 ± 10.88	31.25 ± 7.38
30	44.00 ± 9.53	47.80 ± 10.16	**40.70** ± **11.95**	45.67 ± 10.06	44.80 ± 9.42	44.50 ± 10.45	22.25 ± 4.63
31	45.00 ± 10.35	52.90 ± 11.78	38.20 ± 12.32	38.33 ± 6.61	38.60 ± 10.45	**37.67** ± **10.97**	21.00 ± 3.57
32	49.60 ± 11.01	52.00 ± 10.93	51.60 ± 12.27	**49.17** ± **10.67**	49.60 ± 13.47	49.43 ± 11.99	24.75 ± 4.63
33	48.90 ± 10.80	45.90 ± 10.63	46.20 ± 10.18	51.67 ± 11.24	47.20 ± 10.60	**44.33** ± **12.23**	24.25 ± 5.66
34	44.10 ± 12.02	46.40 ± 9.95	45.60 ± 10.03	45.83 ± 10.35	42.70 ± 9.96	**42.00** ± **7.50**	24.50 ± 3.50
35	18.90 ± 6.87	23.30 ± 8.96	27.70 ± 11.66	25.33 ± 9.91	18.40 ± 8.72	**18.17** ± **9.33**	10.00 ± 2.89
36	47.30 ± 12.50	46.70 ± 9.72	44.90 ± 11.85	48.67 ± 10.82	33.70 ± 12.49	**30.50** ± **10.20**	19.50 ± 6.21
37	26.60 ± 8.30	26.90 ± 9.94	26.00 ± 8.81	29.00 ± 8.65	23.80 ± 8.12	**23.00** ± **10.20**	13.50 ± 6.15
38	53.40 ± 10.66	**51.10** ± **10.02**	51.70 ± 9.35	53.00 ± 9.52	52.40 ± 12.09	51.50 ± 12.12	33.50 ± 3.76
39	28.60 ± 9.26	41.50 ± 10.22	29.80 ± 11.82	37.00 ± 9.25	28.20 ± 11.33	**27.00** ± **9.25**	20.00 ± 6.12
40	48.60 ± 9.32	47.50 ± 10.99	53.10 ± 10.25	**47.17** ± **7.62**	48.40 ± 10.81	48.83 ± 9.16	31.00 ± 6.69
41	25.70 ± 9.04	**10.40** ± **6.05**	19.60 ± 10.39	27.50 ± 7.74	15.90 ± 7.47	14.67 ± 8.80	8.00 ± 2.30
42	56.00 ± 7.95	57.00 ± 10.83	**51.00** ± **10.97**	54.50 ± 8.84	51.10 ± 10.51	51.67 ± 10.11	27.25 ± 6.06
43	6.70 ± 5.31	3.80 ± 4.47	3.60 ± 4.52	**2.00** ± **2.82**	4.90 ± 4.79	4.17 ± 4.17	2.50 ± 2.36
44	10.60 ± 6.11	9.80 ± 6.54	10.70 ± 6.47	17.00 ± 8.37	**8.40** ± **7.45**	10.83 ± 7.55	5.25 ± 3.22
45	46.20 ± 9.18	49.20 ± 10.27	49.40 ± 12.60	55.50 ± 11.62	**45.70** ± **8.45**	47.50 ± 10.97	31.25 ± 8.76
46	30.58 ± 7.46	35.58 ± 9.72	24.92 ± 9.16	31.81 ± 9.44	**24.67** ± **8.07**	25.42 ± 8.71	12.70 ± 5.05
47	27.10 ± 11.21	25.10 ± 8.36	29.10 ± 8.79	30.83 ± 11.07	**22.00** ± **8.02**	25.83 ± 9.83	12.75 ± 5.46
48	44.00 ± 10.69	**11.80** ± **7.68**	19.00 ± 8.45	30.00 ± 8.41	35.30 ± 11.31	21.83 ± 9.69	11.50 ± 5.80
49	10.50 ± 7.16	13.00 ± 6.70	**10.40** ± **4.93**	12.50 ± 8.59	13.70 ± 6.91	12.50 ± 6.66	15.00 ± 6.12
50	0.00 ± 0.00	0.00 ± 0.00	0.00 ± 0.00	0.00 ± 0.00	0.00 ± 0.00	0.00 ± 0.00	0.00 ± 0.00
51	50.40 ± 9.52	**43.10** ± **11.29**	47.10 ± 9.69	45.67 ± 9.89	47.90 ± 11.39	46.83 ± 10.71	27.00 ± 5.11
52	42.50 ± 9.75	49.60 ± 10.59	39.50 ± 9.81	43.00 ± 9.15	40.10 ± 10.86	**38.17** ± **13.10**	19.00 ± 4.89
Average	36.31 ± 9.14	38.46 ± 9.62	33.88 ± 9.38	36.80 ± 9.06	33.23 ± 9.38	**31.81** ± **9.06**	17.78 ± 4.90

A comparison of other statistical measures such as sensitivity, specificity and Cohen’s kappa index is shown in Table 3. The highest values of each statistical measure are highlighted in bold. It can be seen that OPTICAL achieved the highest average sensitivity, specificity and Cohen’s kappa index on both the datasets. This shows that OPTICAL can predict or classify the positive and negative samples with higher accuracies compared to other methods thus achieving the lowest misclassification rate. OPTICAL also achieved the highest Cohen’s kappa index showing that it is more reliable than other competing methods.

**Table 3:** A comparison of the statistical measures of the proposed predictor with other competing methods.

Dataset	Method	Sensitivity	Specificity	Cohen’s kappa index
BCI competition IV dataset 1	CSP	0.694	0.700	0.369
	DFBCSP	0.794	0.795	0.542
	SBLFB	0.806	0.801	0.589
	SFTOFSRC	0.697	0.771	0.296
	OPTICAL*	0.817	0.808	0.615
	OPTICAL	**0.833**	**0.825**	**0.650**
GigaDB dataset	CSP	0.638	0.621	0.297
	DFBCSP	0.619	0.616	0.266
	SBLFB	0.666	0.658	0.339
	SFTOFSRC	0.623	0.531	0.291
	OPTICAL*	0.674	0.662	0.364
	OPTICAL	**0.688**	**0.675**	**0.374**

### Real-time implementation of the proposed predictor (OPTICAL)

Any method developed for improving BCI systems should be such that it can be effectively implemented in real-time and this has been a major issue. In this study, we have used the rest-state together with other non-task related signals of GigaDB dataset to test if the system can perform well in real-time. The one-versus-rest method has been utilized for learning the CSP spatial filters in the real-time implementation as it involves 3 class classification. Firstly, classification is done to classify the non-task related signals against the MI task signals (left hand and right-hand MI tasks). Once this is done, those trials that are classified as MI task signals are then classified as left hand or right-hand MI task signals. Thus, different spatial filters are learned for each of the stages. The results of the real-time (3 class) implementation of OPTICAL are shown in Table 2 labeled as Real-time. We achieved an average misclassification rate of 17.78%, which is very promising. To avoid data imbalance, we randomly selected the number of non-task related trials equal to the total number of MI task related trials. This real-time average misclassification rate is lower compared to the two-class average misclassification rate of 31.81%. This may be because the non-task related EEG signals and task related MI EEG signals are easily separable due to their distinctive characteristics. We also performed the real-time implementation of OPTICAL using multi-class CSP^56^, which requires only one level of classification and obtained an average misclassification rate of 22.88% using GigaDB dataset. Since the one-versus-rest approach performed well compared to the multi-class approach achieving greater than 5% difference in average misclassification rate, we have reported the results of the one-versus-rest approach. In future, experiments will also be carried out by including non-task related MI EEG data to further test the reliability of OPTICAL in real-time. However, the current results are promising and provide key findings for future research work.

## Discussion

Our proposed predictor, OPTICAL, has outperformed all other competing methods. We have shown the results for both OPTICAL* and OPTICAL. OPTICAL* predictor does not perform parameter optimization of the LSTM network as mentioned earlier. On the other hand, the proposed OPTICAL predictor involves optimization of the LSTM network hyper-parameters using Bayesian Optimization. It can be seen from Tables 1 and 2 that optimizing the LSTM network hyper-parameters resulted in a further improvement of the misclassification rate by 1.78% and 1.42% on BCI competition IV dataset 1 and GigaDB dataset, respectively. The DFBCSP and SFTOFSRC approaches did not perform well. While the DFBCSP method had a good performance on BCI competition IV dataset 1, it did not perform well on GigaDB dataset. On the other hand, reasonable performance of the SFTOFSRC method was noted on BCI Competition III dataset IVa and BCI Competition IV dataset IIb (as reported by the authors). However, it did not perform well on both the datasets used in this work and achieved the highest average misclassification rates for both the datasets. The GigaDB dataset has a large number of subjects (52 subjects) and using this dataset shows the reliability and robustness of the approaches. OPTICAL performed well on both datasets showing that it is more reliable and robust predictor compared to other competing methods.

**Figure 4:**
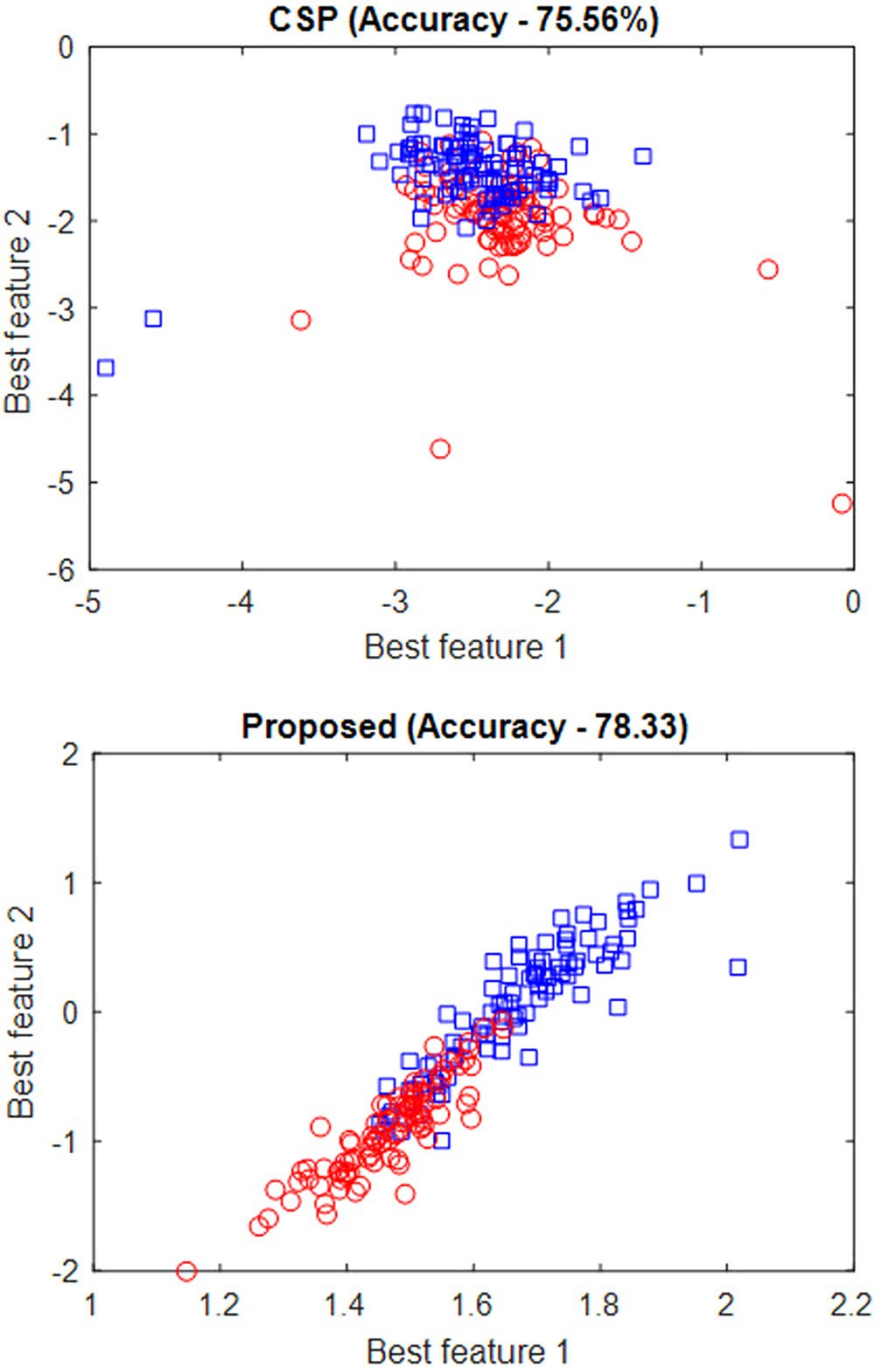
Distribution of the best two features obtained using CSP and proposed predictor (OPTICAL).

The distribution of the best two features obtained using the CSP approach and the proposed OPTICAL predictor for one of the trial runs using subject 01 of GigaDB dataset are shown in Fig. 4. It can be seen that the proposed OPTICAL predictor has features that are more separable, which accounts for the improved performance of OPTICAL.

**Figure 5:**
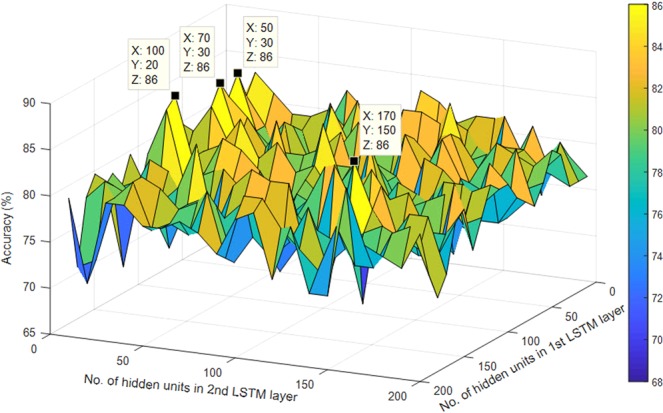
Accuracies of the LSTM network having two LSTM layers with varying number of hidden units obtained using subject *a* of BCI competition IV dataset 1. The x-axis represents the number of hidden units in the 1^st^ LSTM layer, the y-axis represents the number of hidden units in the 2^nd^ LSTM layer and z-axis represents the accuracy.

Moreover, in this study, we have proposed a sliding window approach for obtaining the sequence input for the LSTM network. In doing so, parameters such as the number of LSTM layers, the number of hidden units in each LSTM layer, the length of the sliding window (*l*) and the overlap (*t*) had to be selected. We selected a sliding window length of 25% of the trial length and approximately 5% of trial length as the overlap. These two parameters were chosen such that all sample points in a trial were utilized without the need for padding. Using these parameters, we determined the optimal LSTM network. Experiments were carried out to determine the number of LSTM layers and the number of hidden units in each LSTM layer for optimal performance. We limited our experiments to a maximum of 2 LSTM layers and a maximum of 200 hidden units in each LSTM layer in order to keep the computational complexity of the system low. It was seen that using two LSTM layers produced better results compared to using a single LSTM layer. The result for single LSTM layer network with a different number of hidden units is shown in Fig. S1 (refer to Supplementary Text). The results for the LSTM network having two LSTM layers with a varying number of hidden units in each LSTM layer is shown in Fig. 5. As can be seen from Fig. 5, highest accuracies were obtained at four different combinations. We tested these four combinations on few other subjects for both datasets and the network having 100 hidden units in 1^st^ LSTM layer and 20 hidden units in 2^nd^ LSTM layer performed well compared to the other three combinations. Hence, LSTM network having two LSTM layers with 100 hidden units in 1^st^ LSTM layer and 20 hidden units in 2^nd^ LSTM layer have been used in OPTICAL. Although we have selected the above mentioned hidden units for our proposed predictor, this is not the optimal LSTM network size for all the subjects. To obtain optimal performance for all subjects, we need to optimize the LSTM network size for each of the subjects. This has not been done in this study. We also did not optimize the window length and overlap parameters, which will be considered in future works. Other aspects that will be considered in future works are using other variants of LDA algorithms^57–59^, performing feature selection^60–62^ instead of LDA, and combining two or more methods^63–66^ to further improve the performance of OPTICAL.

**Figure 6:**
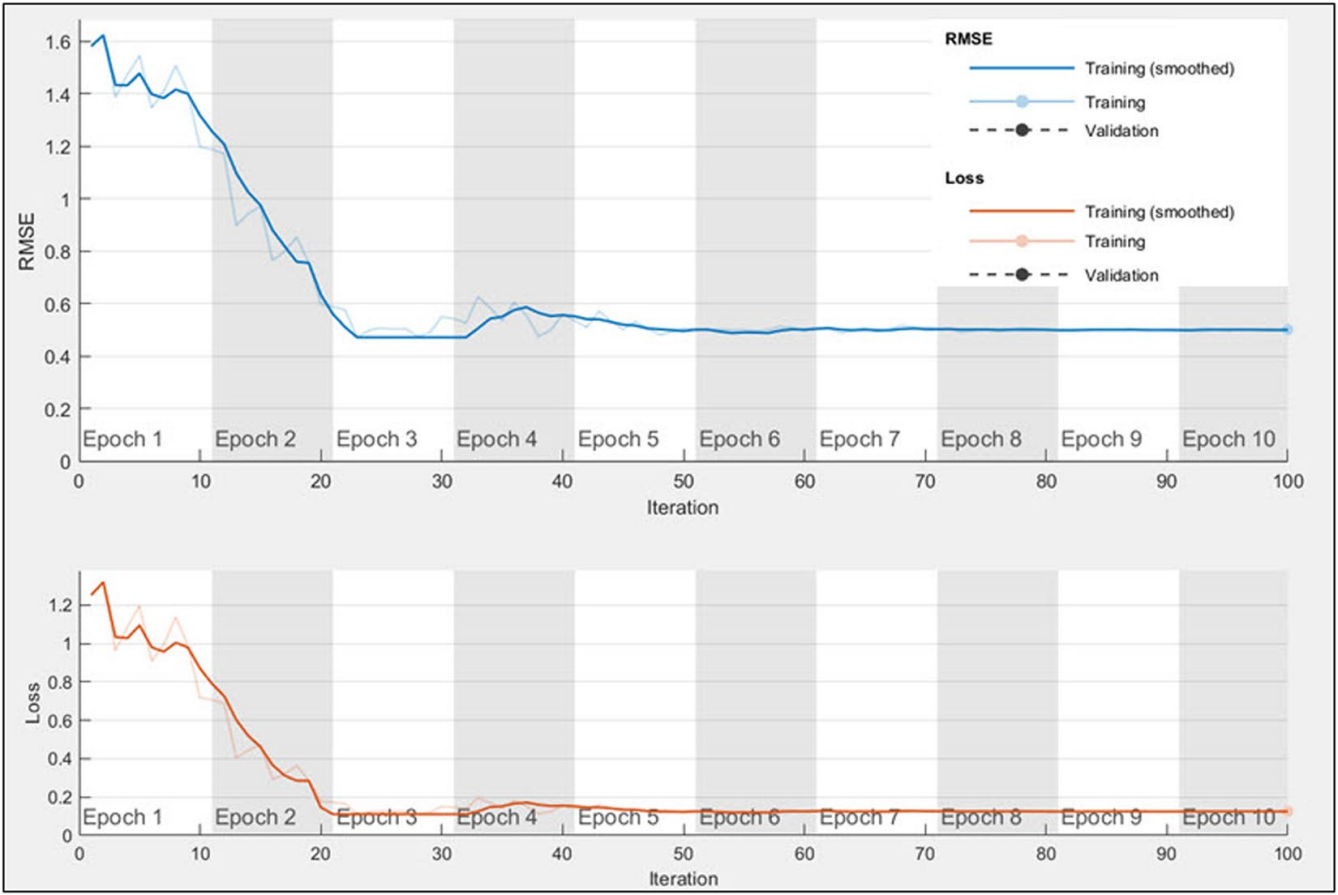
Graph showing how the LSTM network learns the hyper-parameters to minimize the loss function.

The graph of Fig. 6 shows how the root mean squared error and the loss function are minimized during the training of the LSTM network for one of the trial runs of subject 01 of GigaDB dataset. It was noted that OPTICAL can learn the LSTM network in 100 iterations which takes about six seconds. This does not include the time taken to optimize the LSTM network parameters. Similar graphs were obtained for other subjects on both the datasets. As reported in our previous work^38^, the time taken to process and classify a test signal for CSP and SBLFB are 1.00 ms and 4.60 ms, respectively. The time taken to process and classify a test signal using the OPTICAL predictor is 23 ms for the multi-class approach and 37 ms for the one-versus-rest approach, which makes it suitable for real-time applications. OPTICAL achieved improved performance compared to other approaches such as CSP and SBLFB at the expense of increased computational time. It is also worth mentioning that the time taken to classify a test signal using one-versus-rest approach would increase linearly with increasing number of MI tasks that need to be classified. This is due to the fact that for an *n*-class classification problem, the one-versus-rest approach will require *n* − 1 levels of classification. In this work, we considered the 3-class classification problem to evaluate the performance of OPTICAL when implemented in real-time. Therefore, two levels of classification are required where the first level is used to differentiate between tasks related MI and non-task related EEG signals, while the second level of classification is used to differentiate between the two tasks related MI EEG signals. On the other hand, there will be only a slight increase in the time taken to classify a test signal using the multi-class approach as the number of MI tasks is increased. Thus, the choice of using the one-versus-rest approach or the multi-class approach will depend on the specific application where OPTICAL needs to be used. We have also shown that OPTICAL can perform well for real-time classification. Furthermore, to show that the performance improvement achieved by OPTICAL is significant, we have performed paired t-test with a significance level of 5%. This paired t-test was performed using the results of the proposed OPTICAL predictor and the results of SBLFB predictor (the second best performing method). The p-values obtained were 0.025 and 0.005 for BCI competition IV dataset 1 and GigaDB dataset, respectively. This shows that significant improvements have been achieved using OPTICAL for both the datasets.

**Figure 7:**
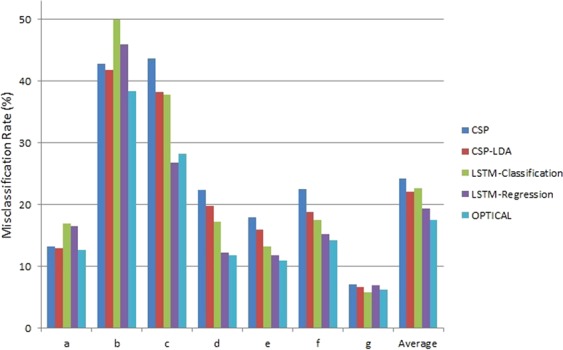
The misclassification rates of different experiments conducted using BCI competition IV dataset 1.

Furthermore, EEG signals have low signal-to-noise-ratio, and as such, the features extracted are also noisy. LDA projects the feature space onto a lower-dimensional space with good class-separability while minimizing the noise. Thus, we have used the reduced dimensional feature space (obtained by performing LDA) as input to the SVM classifier instead of directly feeding the CSP variance based features to the SVM classifier (as done in our previous work^30^). It helps in reducing the noise present in the CSP variance based features and results in an improvement in the classification performance. This is evident from Fig. 7, where it is shown that using the reduced dimensional feature space obtained from LDA as input to the SVM classifier (CSP-LDA) achieved lower misclassification rate compared to directly using the CSP variance based features as input to the SVM classifier.

Moreover, the EEG signal for the same MI task varies between different sessions due to the slight changes in the position of the electrodes. This result in the EEG signals being scaled or offset by some value between different sessions. The classification-based LSTM network uses the softmax layer that is not scale-invariant and may result in degrading the performance of the system. On the other hand, the regression-based LSTM network^67,68^ is scale invariant and helps to tackle this problem to some extent. It can be seen from Fig. 7 that the regression-based LSTM network with SVM classifier performs better than the classification-based LSTM network for EEG signal classification. Therefore, we have employed the regression-based LSTM network.

Furthermore, we combine the power of both CSP and LSTM features. It can be noted that 3 out of the 7 subjects (for BCI competition IV dataset 1) obtained lower misclassification rate using CSP-LDA in comparison to the LSTM-regression based network. Therefore, we have combined the CSP-LDA approach with the LSTM-regression based network (resulting in the proposed OPTICAL predictor), which boosts the performance of the overall system. It can be noted from Fig. 7 that combining these 2 approaches resulted in further reduction in the misclassification rate of 5 out of the 7 subjects while also obtaining the lowest average misclassification rate. Thus, the framework presented in Fig. 1 has been adopted.

To add on, it is also worth noting that the classification performance of the artificially generated data for BCI Competition IV dataset 1 (subjects *c*, *d* and *e*) was good with subjects *d* and *e* achieving less than 12% misclassification rate. These results are in agreement with the results obtained by other related works such as CSP and SBLFB approaches.

Moreover, the hyper-parameters learned by the Bayesian optimization process differed significantly between various subjects. This is the reason why mostly subject-dependent approaches^19,20,23,26,31,34,35,51,52^ have been proposed as the MI EEG signals for the same task varies between subjects. The MI EEG signals of a particular subject may also vary between different sessions due to slight deviation in the exact placement of the sensors. This will require re-tuning of the hyper-parameters, however, this is not practical. Thus, to tackle this problem, covariate shift detection algorithms^69–72^ are recommended in order to correct the shift in the MI EEG data that occurs between different sessions.

## Conclusion

In this study, we have introduced a new predictor called OPTICAL which utilizes optimized CSP and LSTM network for the classification of EEG signals. A sliding window approach has been proposed for solving the problem of obtaining sequence input for the LSTM network. Significant improvements have been achieved on both the datasets, with GigaDB dataset having a considerably large number of subjects showing that OPTICAL is robust and reliable predictor. Promising results have been achieved as OPTICAL outperformed other competing methods in terms of misclassification rate, sensitivity, specificity and Cohen’s kappa index. OPTICAL can be used to develop improved BCI systems. Although we have evaluated OPTICAL using MI EEG datasets, it should also perform well for EEG signal classification in other applications such as sleep stage detection and seizure detection. Future works will consider deeper networks and hybrid approaches by combining OPTICAL with other approaches.

## Supplementary information

Supplementary Material

## Data Availability

The datasets used in this work are publically available for the research committee.
