# Supplementary material for: Brain wave classification using long short-term memory network based OPTICAL predictor
Source: Sci Rep. 2019 Jun 24;9:9153. doi: 10.1038/s41598-019-45605-1 (PMC6591300; doi:10.1038/s41598-019-45605-1)
Supplement: Supplementary file 1 — Supplementary Material [file 41598_2019_45605_MOESM1_ESM.pdf]

Supplement 1 for Brain wave classification using long short-term memory network based OPTICAL predictor

Shiu Kumar[4, 5, *, □], Alok Sharma[1, 2, 3, 4 *, □], Tatsuhiko Tsunoda[2, 3, &]

*Correspondence: shiu.kumar@fnu.ac.fj; alok.sharma@griffith.edu.au
[□]Equal Contributors
[&]Last Author
[1]Institute for Integrated and Intelligent Systems, Griffith University, Brisbane, QLD-4111, Australia
[2]Department of Medical Science Mathematics, Medical Research Institute, Tokyo Medical and Dental University, Tokyo, Japan
[3]Laboratory for Medical Science Mathematics, RIKEN Center for Integrative Medical Sciences, Yokohama, Kanagawa, Japan
[4]The University of the South Pacific, Suva, Fiji
[5]Fiji National University, Suva, Fiji

In this supplementary text, we present the result for the single LSTM layer network in Fig. S1 with varying number of hidden units and their corresponding accuracies. The LSTM network architecture used in our work is presented in more detail. We also present details on how Bayesian optimization works to find the best feasible hyper-parameters (initial learn rate and L2 regularization).

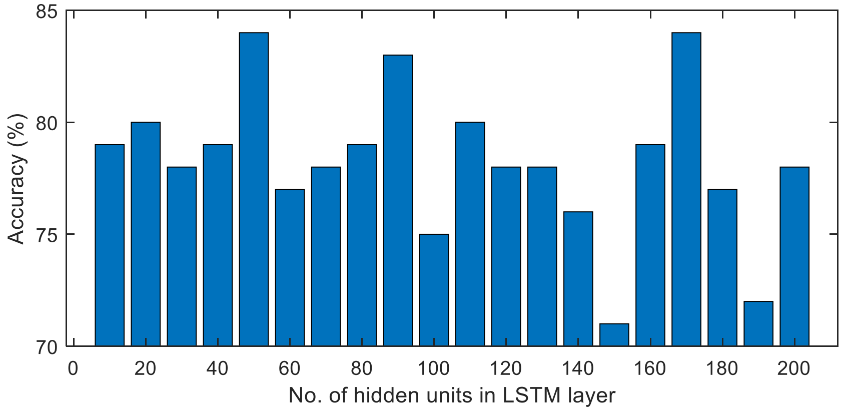

Fig. S1. Accuracies of the LSTM network having one LSTM layer with varying number of hidden units obtained using subject *a* of BCI competition IV dataset 1.

LSTM Network Architecture for regression

The LSTM network architecture used in this work is shown in Fig. S2. The input to the network is a sequence input, followed by 2 LSTM layers, the fully connected layer and regression layer at the output. The sequence input layer is used to provides the time series or sequence input to the network. Long-term dependencies between a series of sequence data is learnt by the LSTM layer. The LSTM layer architecture used in this work is shown in Fig. S3, which shows how the *d*-dimensional sequence input matrix F with a length of *s* flows through the LSTM layer. The $F_{w_j}^{i}$ represents the sequence input where *i* denotes the *i*-th feature obtained from the *j*-th windowed segment of the respective trial (as per sequence input matrix shown in Fig. 2 in the manuscript). The *h* and *c* denotes the hidden/output states and cell states, respectively. The initial state of the network and the first sequence input $F_{w_1}^{i}$ becomes

the input for the 1st LSTM block, which computes the 1st output h_1 and c_1 (updated cell state). The LSTM blocks in between the 1st and last LSTM block takes $F_{w_t}^{i}$ sequence input and current states of the network (c_{t-1}, h_{t-1}) for the t-th LSTM block for computing the updated output state *ht* and the updated cell state *ct*. Information that is learned from previous sequence inputs are contained by the cell states. Information is added or removed (update controlled using gates) from the cell states at each step of the sequence input.

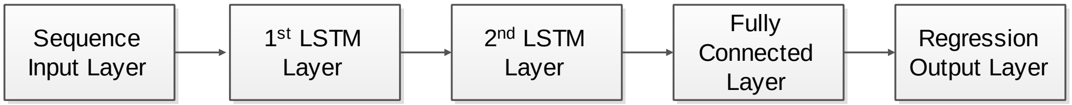

Fig. S2. The LSTM network architecture of the proposed GA-CSP-LSTM predictor.

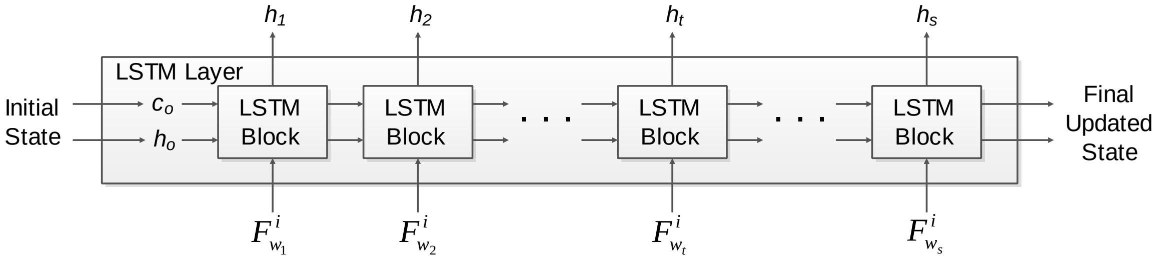

Fig. S3. The LSTM layer architecture used for proposed GA-CSP-LSTM predictor.

In general, the LSTM architecture comprises of a memory cell, an input gate, a forget gate and an output gate. The memory cell of the LSTM layer stores or remembers values (states) for either long or short times. On the other hand, the degree to which a new information or value flows into the cell of a LSTM layer is controlled by the input gate, the degree to which an information or value remains in the cell of the LSTM layer is controlled by the forget gate while the degree to which information or value stored in the cell of the LSTM layer is utilized for computing the output activation is controlled by the output gate (This paragraph is included in the manuscript). Fig. S4 shows how the data flows through the LSTM block for the t-th step of the input sequence. The input weights $\boldsymbol{W}$, recurrent weights $\boldsymbol{R}$ and bias $\boldsymbol{b}$ are the weights that are learned by the LSTM layer.

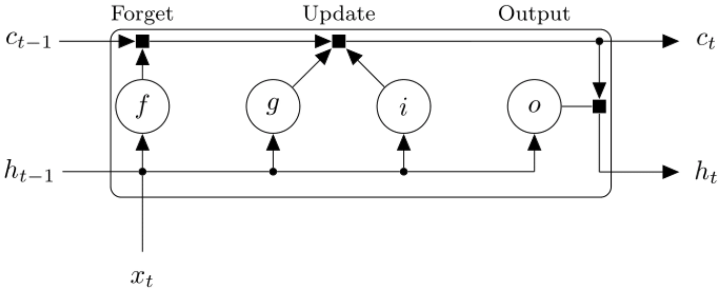

Fig. S4. Diagram showing the flow of data through the LSTM block for t-th step of the input sequence[1].

The cell state at step t of the input sequence is calculated by $c_t = f_t \cdot c_{t-1} + i_t \cdot g_t$ while the hidden/output state is calculated by $h_t = o_t \cdot \sigma_c(c_t)$, where *i*, *f*, *g* and *o* denotes the input gate, forget gate, cell candidate and hidden/output gate, respectively. The (·) represents the element-wise multiplication (Hadamard product operator) and σ_c denotes the state activation function. Each of the components at sequence input t is calculated as follows:

$$i_t = \sigma_g(W_i x_t + R_i h_{t-1} + b_i)$$

$$f_t = \sigma_g(W_f x_t + R_f h_{t-1} + b_f)$$

$$g_t = \sigma_c(W_g x_t + R_g h_{t-1} + b_g)$$

$$o = \sigma_g(W_o x_t + R_o h_{t-1} + b_o)$$

The tangent hyperbolic function has been used as the state activation function, $\sigma_c(x) = \tanh(x)$ while the sigmoid function is used as the gate activation function, $\sigma_g(x) = (1 + e^{-x})^{-1}$.

Selection of hyper-parameters of the LSTM network using Bayesian Optimization

The LSTM network used in this work has been optimized using Bayesian optimization that is utilized for selecting the best feasible hyper-parameters of the selected LSTM network. The objective function $f(x)$, which in this case is the 10-fold cross validation error, is minimized using Bayesian optimization for the hyper-parameters within the specified bounded domain. The Bayesian optimization algorithm computes the objective function values $y_i = f(x_i)$ for a number of randomly selected points within the bounds of the specified hyper-parameters. The log-scaled probability distribution of each component has been used. The Gaussian model of $f(x)$ is updated and the posterior distribution over functions $Q(f|x_i, y_i\, for\, i = 1, \ldots, t)$ is obtained. Based on the Gaussian process model of f the acquisition function $a(x)$ is maximized in order to determine the next evaluation point[2]. Depending on the posterior distribution function Q, the acquisition function evaluates how good a point x is for the given network. The above procedure is repeated until the stopping criterion is reached. The stopping criterion used in this work was that either maximum number of iterations reached 25 or the time taken for the Bayesian optimization reached 5 minutes, whichever occurred first. The best feasible points determined were then used train the LSTM network using training data. In this work, we have used MATLAB for all processing and the Bayesian optimization algorithm package available in MATLAB has been utilized.

Results of real-time implementation using multi-class approach

Table S1 shows the results obtained for real-time implementation using multi-class approach (for GigaDB dataset), where multi-class CSP is utilized. The average misclassification rate is 22.88% ± 4.94.

Table S1: Misclassification rate (%) for real-time implementation of OPTICAL using the multi-class approach (GigaDB dataset). No. represents the subject number.

No.	Misclassification rate	No.	Misclassification rate	No.	Misclassification rate	No.	Misclassification rate
1	21.00 ± 5.16	14	3.50 ± 4.28	27	30.50 ± 4.05	40	26.75 ± 5.01
2	31.25 ± 3.39	15	26.25 ± 5.92	28	28.50 ± 7.19	41	24.00 ± 6.03
3	7.25 ± 5.95	16	27.00 ± 4.68	29	28.25 ± 3.92	42	33.75 ± 6.15
4	24.50 ± 7.53	17	26.50 ± 4.89	30	27.75 ± 4.63	43	6.00 ± 2.42
5	0.50 ± 1.05	18	26.00 ± 4.12	31	25.50 ± 3.69	44	8.75 ± 3.95
6	11.50 ± 3.94	19	29.50 ± 5.24	32	25.00 ± 7.17	45	31.75 ± 5.14
7	31.88 ± 7.55	20	24.75 ± 1.42	33	25.50 ± 6.85	46	25.00 ± 5.01
8	27.75 ± 4.92	21	27.25 ± 4.16	34	33.00 ± 7.62	47	25.25 ± 4.63
9	37.29 ± 6.32	22	23.00 ± 5.37	35	11.50 ± 5.03	48	23.25 ± 4.57
10	23.25 ± 6.13	23	29.50 ± 6.21	36	22.75 ± 9.16	49	5.25 ± 2.99
11	28.25 ± 4.57	24	24.50 ± 5.75	37	26.25 ± 2.95	50	0.00 ± 0.00
12	24.25 ± 5.41	25	27.00 ± 5.37	38	30.50 ± 7.71	51	29.00 ± 3.57

13	22.50 ± 3.54	26	1.50 ± 2.11	39	19.50 ± 5.87	52	29.25 ± 6.57

Reference

1 *Long Short-Term Memory Networks*, <https://au.mathworks.com/help/deeplearning/ug/long-short-term-memory-networks.html> (2018).

2 *Bayesian Optimization Algorithm*, <https://au.mathworks.com/help/stats/bayesian-optimization-algorithm.html> (2018).
